# ^18^F-FDG PET/CT in Relapsed Endometrial Cancer Treated with Preoperative PD-1 Inhibitor Dostarlimab

**DOI:** 10.3390/diagnostics11081353

**Published:** 2021-07-28

**Authors:** Romain-David Seban, Anne Donnadieu, Gabrielle Journo, Francois-Clement Bidard, Capucine Richard, Roman Rouzier, Laurence Champion

**Affiliations:** 1 Department of Nuclear Medicine and Endocrine Oncology, Institut Curie, 92210 Saint-Cloud, France; capucine.richard@curie.fr (C.R.); laurence.champion@curie.fr (L.C.); 2 Laboratoire d’Imagerie Translationnelle en Oncologie, Inserm, Institut Curie, 91401 Orsay, France; 3 Department of Medical Oncology, Institut Curie, PSL Research University, 92210 Saint-Cloud, France; anne.donnadieu@curie.fr (A.D.); francois-clement.bidard@curie.fr (F.-C.B.); 4 Department of Medical Imaging, Institut Curie, 92210 Saint-Cloud, France; gabrielle.journo@curie.fr; 5 Circulating Tumor Biomarkers Laboratory, SiRIC, Institut Curie, PSL Research University, 75005 Paris, France; 6 Department of Surgery, Institut Curie, PSL Research University, 92210 Saint-Cloud, France; roman.rouzier@curie.fr

**Keywords:** ^18^F-FDG PET/CT, endometrial adenocarcinoma, dostarlimab, immunotherapy, tumor response, microsatellite instability high/hypermutated

## Abstract

Dostarlimab is an immune checkpoint inhibitor (ICI) targeting the Programmed-Death-1 (PD-1) co-receptor, recently approved by the European Medicines Agency (EMA) and the Food and Drug Administration (FDA) as a novel therapy for recurrent or advanced endometrial cancer. We report the case of a 64-year-old woman, experiencing vaginal recurrence with microsatellite instability high/hypermutated of a FIGO stage IA grade 2 endometrial endometrioid adenocarcinoma. She received preoperative chemotherapy with four cycles of carboplatin plus paclitaxel, with stable disease on pelvic magnetic resonance imaging (MRI) and fluorine-18 fluorodeoxyglucose positron emission tomography (^18^F-FDG PET/CT). Dostarlimab (500 mg intravenously every 3 weeks) was then introduced. The subsequent evaluation after three perfusions demonstrated a complete metabolic response on ^18^F-FDG PET/CT according to immunotherapy-modified PET response criteria in solid tumors (imPERCIST) criteria, then confirmed by MRI according to immune response evaluation criteria in solid tumors (iRECIST). This clinical description suggests that ^18^F-FDG PET/CT might take place among available tools for guiding the preoperative management for recurrent endometrial cancer patients receiving dostarlimab immunotherapy that should be further explored through clinical trials.

In this case (Figure 1), ^18^F-FDG PET/CT highlighted the tumor resistance to platinum-based chemotherapy and it also revealed its remarkable sensitivity to immunotherapy with dostarlimab. This rare description highlights the utility of ^18^F-FDG PET/CT for guiding the preoperative management for recurrent endometrial cancer patients using PET-based immune-related response criteria [1,2].

The very recent literature reports that dostarlimab monotherapy (TSR-042) is associated with significant antitumor activity and promising response rates for relapsed endometrial cancer patients [3,4], especially those with deficient mismatch mutation repair (MMR) [5] or MSI-H [6].

Given this update on the evolving role of immunotherapy as treatment of patients with recurrent endometrial cancer [7,8], we strongly believe that ^18^F-FDG PET/CT might take a place among available tools for a reliable strategy to manage preoperative therapy and should be further explored through clinical trials evaluating the clinical benefit with dostarlimab.

## Figures and Tables

**Figure 1:**
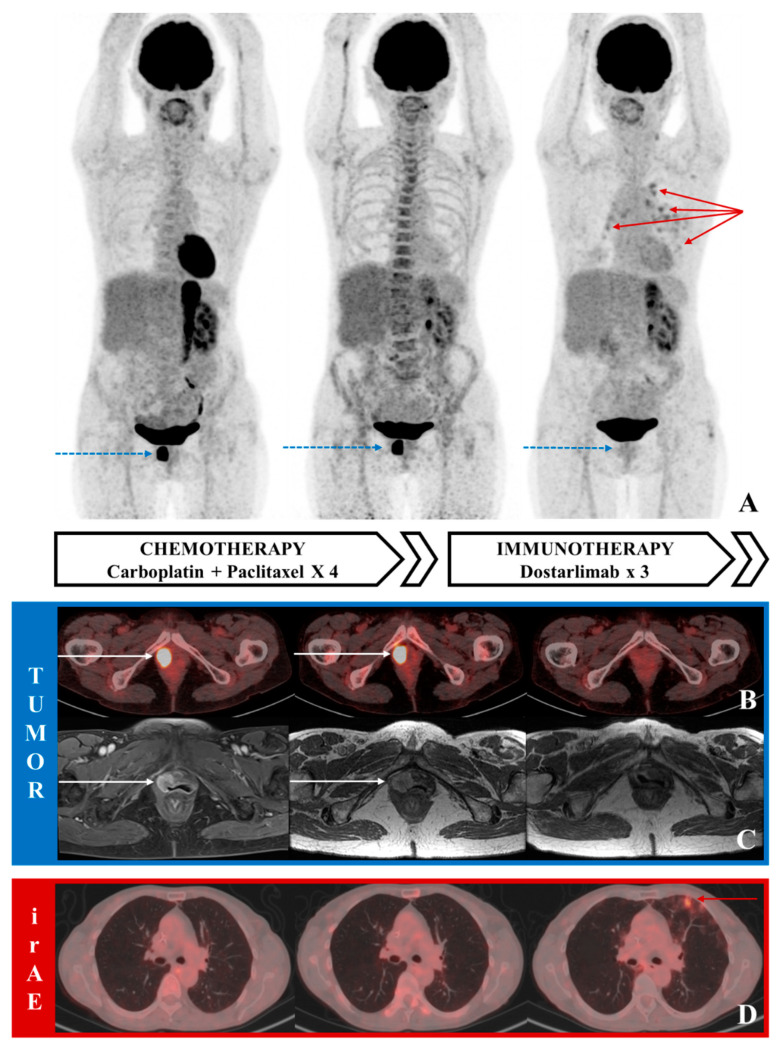
^18^F-FDG PET/CT images and axial T1 gadolinium-enhanced pelvic MRI (with fat-saturation for the left image and without for the two others, because fat-saturation technique was not available/performed) at recurrence and follow-up during chemotherapy and immunotherapy showing pathological FDG uptake (**A**: maximum intensity projection-MIP PET images; **B**: fused axial PET/CT images) and contrast enhancement (**C**) corresponding to the tumor lesion in the vagina (blue arrows on the MIP, white arrows on ^18^F-FDG PET/CT and MRI images), without any metabolic or morphologic changes under chemotherapy. After three injections of dostarlimab, a complete response was observed both on pelvic MRI and ^18^F-FDG PET/CT, associated with irAE (immune-related adverse event) pneumonitis, suggested by hypermetabolic peripheral reticular markings (red arrows on the MIP and ^18^F-FDG PET/CT images) and small ground-glass opacities (**D**), then confirmed by bronchoalveolar lavage.

## Data Availability

The data presented in this study are available on request from the corresponding author.
